# Radical embodied cognitive neuroscience: addressing “grand challenges” of the mind sciences

**DOI:** 10.3389/fnhum.2014.00796

**Published:** 2014-10-07

**Authors:** Luis H. Favela

**Affiliations:** ^1^Department of Philosophy, University of CincinnatiCincinnati, OH, USA; ^2^Department of Psychology, Center for Cognition, Action, and Perception, University of CincinnatiCincinnati, OH, USA

**Keywords:** data deluge, dynamical systems theory, radical embodied cognitive neuroscience, self-organized criticality, theoretical modeling

## Abstract

It is becoming ever more accepted that investigations of mind span the brain, body, and environment. To broaden the scope of what is relevant in such investigations is to increase the amount of data scientists must reckon with. Thus, a major challenge facing scientists who study the mind is how to make big data intelligible both within and between fields. One way to face this challenge is to structure the data within a framework and to make it intelligible by means of a common theory. Radical embodied cognitive neuroscience can function as such a framework, with dynamical systems theory as its methodology, and self-organized criticality as its theory.

## Introduction

The National Science Foundation recently identified “grand challenges” in brain mapping (National Science Foundation, 2013). One of the grand challenges in understanding the brain is the need for a common theoretical language for describing the brain across multiple scales of inquiry (He et al., 2013). A related grand challenge is the need to reduce “Big Data” to “Small Data” (He et al., 2013). The call for a common theoretical language across scales and for data reduction are responses to the massive amounts of data being produced. Government-funded projects such as the BRAIN Initiative (National Institutes of Health, 2014) and the Human Brain Project (European Commission, 2013) produce enormous amounts of data related to brain structure and function. The creation of an ultrahigh-resolution 3-D model of a single human brain, for example, is speculated to result in about 21,000 terabytes of data (Amunts et al., 2013). The field of neuroscience is starting to experience information overload (Gallagher and Appenzeller, 1999). I refer to this as the “data deluge challenge.”

It should come as no surprise that neuroscience could be reaching a time when it is generating more data than it can handle. Signs of the data deluge challenge date at least as far back as the early 1990s, when neuroscience was described as “data rich, but theory poor” (Churchland and Sejnowski, 1992, p. 16). Neuroscience is now in “urgent need” (Sporns, 2011a) of a common theoretical language and solid theoretical foundation if the accumulated data is to facilitate understanding of the brain, cognition, and behavior.

With the increasing acceptance that investigations of mind span the brain, body, and environment, the grand challenges of identifying a common theoretical language and the urgent need for a solid theoretical foundation are no longer confined solely to neuroscience. Moreover, by accepting that mind spans brain, body, and environment, the other grand challenge of reducing big data to small data potentially becomes one of reducing *enormous* data to small data. These two grand challenges are made far more challenging when they are no longer confined to one scientific field of inquiry, but span multiple fields. Can a cognitive scientist incorporate her motion-capture data with a neuroscientist's connectome data, and can a psychologist incorporate those data in her studies of social networks? One way to face these challenges is to structure the data within a framework and to make it intelligible by means of an underlying theory. *Radical embodied cognitive neuroscience* (RECN) can function as such a framework and self-organized criticality (SOC) can be the theory.

In responding to the data deluge challenge, I begin with a description of a version of radical embodied cognitive neuroscience. Second, I discuss SOC. Next, I present an early attempt at applying RECN across various scales of inquiry. Last, I discuss how the framework and theory can be utilized to address the NSF's grand challenges.

## The framework: radical embodied cognitive neuroscience

### The inspiration: radical embodied cognitive science

The version of RECN that I propose is inspired by Chemero's radical embodied cognitive science (RECS) (2009, 2013). Chemero places his RECS in a lineage of modern psychology that originates in Darwinism, Jamesian functionalism, and Gibsonian ecological psychology (Chemero, 2013). The two main features of Chemero's RECS that are most pertinent to RECN are that it takes ecological psychology as the starting point for its theoretical basis and dynamical systems theory (DST) as its methodology.

Gibson's ecological psychology (1966/1979) contrasts with more mainstream cognitive science and psychology in its investigation of “perceptual systems” (Gibson, 1973/1983) and the underlying commitments that frame these investigations (cf. Fodor and Pylyshyn, 1981; Turvey et al., 1981). Ecological psychologists generally agree that perception is for guiding action and is direct in that the relation between the organism and environment is unmediated by mental representations. Instead of indirect, mental representations, perception is of affordances, which are directly perceivable opportunities for action (Richardson et al., 2008; Chemero, 2009). In order to investigate organism-environment systems, ecological psychologists often utilize DST (e.g., Kugler et al., 1980). Much research consistent with and part of the ecological tradition often involves modeling changes over time in the perception-action cycle of perceptual systems (e.g., Smith and Thelen, 2003). DST utilizes methods from calculus to capture changes of variables over time within a system. What counts as a “system” is not limited to the skin, scales, feathers, or fur boundaries of an organism. A system can be comprised of an organism-environment and their interactions (Beer, 1995; van Gelder, 1995). Taken together, ecological psychology and DST are the core of Chemero's RECS, and provide him with a background theory and methodology. This brief summary of RECS is intended to serve as the launching point for my description of RECN. In the next two sections I present RECN by comparing and contrasting it with RECS.

### The “radical,” the “embodied,” and the “cognitive”

In the currently proposed version of RECN, “radical” refers to the rejection of the “mind as computer” metaphor and the central role of mental representations in guiding actions. “Embodied” refers to the non-brain-centric treatment of cognition. “Cognitive” refers to the target of investigation within this framework. Like RECS (Chemero, 2009, 2013), RECN's use of “cognition” is consistent with the Jamesian functionalist tradition, which treats cognition in terms of meaningful, systematic, often goal-directed, behavior. This loose definition is in no way intended to provide the necessary and sufficient conditions of cognition. Adherents to the commitments of mainstream cognitive science and psychology will not find their “holy grail” here, namely, the mark of the cognitive (cf. Adams and Aizawa, 2008; Menary, 2010). This sense of cognition is more in-line with “mindedness,” whereby cognition begins with perceptual capacities that guide action, and is always involved in an organism's being-in-the-world (cf. Thompson, 2007; Anderson, 2009). This shift from a Cartesian notion of cognition as consisting of a homunculus-like, little person in the head, isolated phenomenon that occurs in brains, to a phenomenon that spans multiple scales across brain, body, and environment, has consequences for how the mind sciences conceive of their focus of inquiry. Thus, for the remainder of this work, I will use “cognition” and “mind” interchangeably.

RECN distinguishes itself from mainstream cognitive science, psychology, and neuroscience in a number of ways. First, although it is fair to say that all contemporary scientific investigations of the mind are physicalist (materialist, naturalist, etc.), there is often a residual Cartesian commitment exhibited by practitioners. Solymosi (2011) refers to this as the “Cartesian materialism” evident in methodological and theoretical commitments to the brain as identical to mind, or the brain as the functional locus of mind. There is no doubt that the brain is essential for most cognitive and behavioral activities. However, RECN does not treat the brain as the central target of investigation when researching mind (cf. Van Orden et al. notion of the “blue-collar brain”; Van Orden et al., 2012).

Second, instead of investigating formal computations acting over mental representations, or the neural implementation of said computations and representations, RECN investigates systems. RECN takes lessons from Gibsonian ecological psychology and treats cognition as something that systems do. Cognition is not an all or nothing phenomenon. There are degrees of cognition. Cognition is something that is realized in organism-environment systems. Depending on what aspect of cognition one investigates, the brain may play a more central role in the explanation, but the body might in another, or, for that matter, the environment outside the skin, scales, feathers, or fur boundaries of an organism. Regardless of what part of the system is focused upon in a particular investigation, all parts of the system are involved. In RECN, the boundaries of cognition can be given pragmatically motivated borders in order to facilitate scientific investigation. The bounds of cognition are not knowable a priori. The bounds of cognition must fit in a theoretical and methodological framework that gels with the empirical evidence. RECN is “embodied” in that cognition is not brain-centric, but spans the brain, body, and environment.

Like its predecessor, RECN utilizes the tools of DST in order to investigate cognition. DST provides both the terminology and techniques for modeling changes in systems over time. DST and its applications to the cognitive, neural, and psychological sciences are not without its challenges. Methodological challenges include the distinction between dynamical and non-dynamical models, properly characterizing the variables, and being clear to distinguish such data as those obtained from the simulation or model, and those from the empirical data (Gelfand and Engelhart, 2012). Theoretical challenges include the explanatory power of dynamical models (Kaplan and Bechtel, 2011) and the compatibility of DST with such concepts as representation, computation, and information processing (Eliasmith, 2012). Despite these challenges, the methods remain powerful and broadly applicable. The virtues and vices of applying DST to investigations of cognition have been addressed in great detail elsewhere and go beyond the scope of the current work (see Haselager et al., 2003; Thelen and Smith, 2006; Bechtel and Abrahamsen, 2010; Stepp et al., 2011; Silberstein and Chemero, 2013). For a defense of DST in investigations of cognition, I refer readers to Chemero (2009) and Thelen and Smith (1994).

Thus far, RECN is mostly indistinguishable from RECS. First, both are “radical” in their rejection of commitments to mental computations and representations as necessary theoretical postulates for explaining cognition. Second, both are “embodied” in their commitment to non-brain-centric treatments of cognition and the investigation of organism-environment systems. Third, both have returned to psychology's Jamesian roots and the investigation of mindedness. Finally, both utilize the tools and methods of DST. In the following section, I begin to differentiate RECN from RECS by describing the “neuroscience” portion of RECN.

### The “neuroscience”

Chemero suggests the possibility of a single scientific framework of the entire brain-body-environment system (2009, p. 153), possibly comprised of work in the ecological and enactive cognitive sciences. Though, he cautions that much more work needs to be done in both of these sciences before they can unite. From this discussion, it can be supposed that Chemero believes that investigations of behavior typical to ecological psychologists will not be enough to fully investigate cognition. On this point, I think he is right. However, I do not think he is correct about the present state of the relevant neural sciences. The cognitive, neural, and psychological sciences are currently poised to comprise a framework for the investigation of brain-body-environment systems. If unified under the heading of “radical embodied cognitive neuroscience,” there is research currently being conducted that could comprise such a framework, research that will be discussed below. Like RECS, RECN would apply the methods of DST. However, RECN would have a different guide to discovery.

To date, DST has been applied in research that spans a variety of mind-related disciplines. Examples include, but are far from limited to, research on single neuron activations (e.g., Izhikevich, 2007), neural networks (e.g., Sporns, 2011b), quantifying consciousness (e.g., Balduzzi and Tononi, 2008), and accounts of mental illnesses such as schizophrenia (e.g., Loh et al., 2007). Although short, this list motivates the claim that DST has been successfully applied to phenomena paradigmatically thought of as causally related to or constitutive of cognition, such as neural activity and consciousness. The same is true of DST in cognitive science and psychology. Accordingly, DST is poised to serve as a shared terminology and methodology across the various cognitive, neural, and psychological sciences. This is why DST is the chosen methodological framework of the current version of RECN, and is a major reason why RECN is similar to RECS. RECN and RECS differ in two major ways though. First, they differ in terms of the emphasis placed on the role of neural accounts of cognition. Second, they differ in regard to their main theoretical commitment, which in turn informs their main guide to discovery.

Chemero's RECS places Gibsonian ecological psychology at the center of its theoretical commitments, especially Gibson's lesson that when investigating cognition, one ought to look at the organism-environment system and not the organism qua entity independent from the world. RECS also places affordances in a prominent position. According to Gibson's ecological theory of perception-action (1966/1979, 1973/1983), perception is of affordances. Although there are a number of competing definitions of the term “affordances” (e.g., Gibson, 1973/1983; Turvey, 1992; Stoffregen, 2003; Chemero, 2009), it is generally agreed among ecological psychologists that affordances are “directly perceivable, environmental opportunities for behavior” (Chemero, 2009, p. 23). These opportunities for behavior are based on properties of the environment and properties of the animal (Stoffregen, 2003). The affordance “pass-through-ability,” for example, is based on the width of the opening of apertures, such as doors, relative to an organism's width (Warren and Whang, 1987; Favela et al., 2014). The investigation of such affordances is central to the ecological psychologist's research program, and it is central to RECS as well.

Although both RECS and RECN share in the Gibsonian commitment to the investigation of organism-environment systems, RECN does not share the commitment to researching affordances. Ecological psychologists tend to research affordances, and affordances tend to be analyzed at the scale of whole organisms. Although there is no in-principle reason why a theory of affordances cannot have a significant neural portion (e.g., Cisek, 2007), ecological psychologists do not investigate the brain. For ecological psychologists, affordances are phenomena that happen at the intersection of organism-environment interactions. Nonetheless, neither the ontological nor epistemological status of affordances is essential to RECN. Thus, RECN can be committed to the investigation of organism-environment systems without being committed to a Gibsonian theory of affordances. So, what role does the brain play in RECN?

RECN includes “neuroscience” in the sense that the brain, as part of an organism, is essential to understanding the mind as a systems phenomenon. The affordance guided research of ecological psychology and RECS focus investigations of mind at the intersection of organism and environment. Thus, an account of perception-action can be provided at more overt levels of behavior. RECN wants the best of both worlds: RECN treats organism-environment interactions as essential to explanations of various capacities and features of mind; and like the neurosciences, RECN treats the brain and nervous system as essential, both causally and constitutively, to explanations of various capacities and features of mind. Science is a human enterprise, and humans are limited in how much they can understand at a particular moment (cf. Bechtel and Richardson's discussion of psychological heuristics in scientific theorizing; Bechtel and Richardson, 1993/2010). Since mind spans brain-body-environment, research must be pragmatically motivated in order to get a grip on particular aspects of the system (cf. Sporns, 2012). In some investigations of mind, an account at the scale of organism-environment will be appropriate, but for others, the account must include features at the neural scale. RECN does not emphasize researching affordances because it has a different guide to discovery, one that does not limit investigations to the organism-environment scale. RECN utilizes the theory of SOC as its guide to discovery.

## The theory: self-organized criticality

In the late 1980s, Bak et al. (1987, 1988) proposed SOC as an abstract, general theory of the apparent ubiquity of power laws in nature (Bak et al., 1987, p. 381). Power laws arise near critical points that are found at second-order phase transitions. Second-order phase transitions refer to continuous changes at phase-transition points, where, unlike first-order phase transitions, two phases do not simultaneously exist; there is only one phase at a point (Bar-Yam, 1997, pp. 87–89). SOC was also postulated as a unifying theory for the many phenomena in nature and in the laboratory that demonstrate spatial features such as scale invariance and self-similar structure, as well as temporal dynamics characterized by 1/*f* signals. Self-organization is appealed to as an explanation of how and why systems can be near critical states in so many conditions and substrates (cf. Song et al., 2005). Self-organization refers to the state of some nonequilibrium, dynamic systems to develop structures and patterns of behavior over time without the control of an external agent or central processor (Jensen, 1998). A critical state occurs in a system when, on average, activity of one feature of the system leads to one additional activity, such as one neuron activating another neuron (Beggs and Plenz, 2003, p. 11174) or one nuclear fission event leading to one other fission event (Cutnell and Johnson, 2009, p. 1008). A subcritical state occurs when, on average, one activity leads to less than one subsequent activity. A supercritical state occurs when, on average, one activity leads to more than one subsequent activity. Self-organization and criticality make SOC amenable to the study of complex systems, where “complex” refers to the tendency of systems to exhibit behavior resulting from many components, and their interactions to be placed in critical states.

An early example in the SOC literature is the sand pile model (Bak et al., 1987). Imagine the creation of a pile of sand with additional grains of sand slowly added. At first, the pile continues to grow in a cone-like shape. However, after some time, the pile will be in a critical state whereby an avalanche will occur and the grains of sand tumble down, widening the base of the structure and facilitating the ability of the structure to maintain a higher center point. If more sand is added to the pile, then it will continue to grow again until it reaches another critical state and experiences another avalanche, again widening the base and allowing the center to be higher. If the slope of the pile were measured before each avalanche, a scale-free, or power law distribution will be evident. “Scale-free” refers to statistically, self-similar structures or patterns at varying spatial or temporal scales. An important consequence of this feature is that although the exact location or number of grains of sand that will cause an avalanche cannot be predicted a priori, the probability that a particular location and number of grains of sand will cause an avalanche will be correlated over wide ranges of spatial and temporal scales (Bak et al., 1988, p. 364). That spatial and temporal properties are correlated over wide ranges is indicative of a “cooperative effect” (Bak et al., 1988, p. 364). Such cooperative effects can be understood as the result of a system's being self-organized. Systems are self-organized when there is a reciprocal relationship among local areas and behavior at the global state of the system (Strogatz, 1994; Kelso, 1995). Moreover, such reciprocal relationships often display scale-free, self-similar structures.

Fractals are examples of scale-free, self-similar structures (Mandelbrot, 1977/1983). A fractal is a spatial or temporal structure whereby the global structure is maintained at various scales of observation. Examples of fractal spatial structures include coastlines and mountain ranges, Sierpinksi triangles, tree branching, and cauliflower. Examples of fractal temporal structures include finger tapping (Kadota et al., 2004; Kello et al., 2007), heartbeats (Peng et al., 1995), human gait patterns (Hausdorff et al., 1995), functional magnetic resonance imaging signal changes (Lee et al., 2008), and simple reaction time tasks (Van Orden et al., 2005). The scale-free, self-similar structure of fractals can be quantified by power laws and captured utilizing a number of analytic techniques, such as detrended fluctuation analysis (Ihlen, 2012). If power laws, particularly in the 1/*f* range, are revealed in the analyses, then the results can be interpreted to be indicative of such features as interaction-dominance (Richardson and Chemero, 2014). Although these analytic methods are still being developed and refined (e.g., Ihlen and Vereijken, 2010), and although artificial systems can be created in the lab that display 1/*f* power law features (e.g., Wagenmakers et al., 2005), given the ubiquity of such features in natural systems and the mounting empirical evidence, it is a reasonable hypothesis that something along the lines of SOC is responsible for the generation and maintenance of these spatial and temporal properties.

Since Bak and colleagues first proposed it in the late 1980s, SOC has been utilized to characterize the behavior of various systems, such as rice and sand piles (Bak, 1996), earthquakes (Bak et al., 2002), and the Earth's magnetosphere (Consolini, 2002). Various features of brains have demonstrated SOC: Human brain oscillations (Poil et al., 2008), network connections (Chialvo, 2004; Sporns, 2011b), networks of cortical neurons (Beggs and Plenz, 2003; Pasquale et al., 2008), and spontaneous cortical activity *in vivo* in cats (Hahn et al., 2010) and monkeys (Petermann et al., 2009). Experimental results also indicate that SOC is a ubiquitous feature of more overt scales of cognition and behavior: Interpersonal coordination (Coey et al., 2012), mental image rotation (Gilden, 2001), and stride and gait (Hausdorff et al., 1995).

Thus far, two facts can be said about SOC. First, self-organization and criticality regularly occur together in nature. Second, there is accumulating experimental evidence for SOC in various systems, especially related to the brain, cognition, and behavior. If SOC is the guide to discovery for RECN, then how is it deployed in scientific practice? SOC is deployed in terms of a particular set of theoretical commitments that guide research. Popper once noted that:

Observation is always selective. It needs a chosen object, a definite task, an interest, a point of view, a problem. And its description presupposes a descriptive language […], which in its turn presupposes interests, points of view, and problems (Popper, 1963/2002, p. 62).

In line with this view of scientific practice, RECN treats systems as the chosen object of investigation, with the definite task of explaining how those systems act. The descriptive language of RECN is DST and the point of view is from SOC. Consequently, for the practitioner of RECN, the idea is to look at systems and to explain their behavior in terms of self-organization and critical phase transitions.

What counts as a “system” depends on the research question. What remains the same across various research questions is the idea that systems do not require an external force to drive their behaviors. SOC describes the undirected occurrence of critical dynamics in complex systems ruled by internal interactions (Bak et al., 1988; Jensen, 1998; Rubinov et al., 2011). The notion of “critical states,” as evidenced by power law and self-similar spatial and temporal structures, introduces a third type of behavior for empirical investigation. As Van Orden et al. (2011) note, before complexity science, variation in repeatedly measured values was divided into the categories of regular or random change (p. 640). SOC provides a theory for labeling a third category of states that are neither regular nor random. The sand pile is an example of a state that is neither regular nor random. As discussed above, the behavior of the sand pile is not deterministic, but it is statistically stable. Many nonequilibrium systems can be characterized as being neither regular nor random (Prigogine and Nicolis, 1977). Mind is treated as falling into this third category of behavior. When mind is categorized as a self-organized and critical system, it is said to exhibit the following qualities: It spans brain, body, and environment; it is a self-organized system that is not directed by either an external or internal controller; and it resides in nonequilibrium states that are in constant flux and exhibit self-similar properties at various spatial and temporal scales. When a system exhibits self-organization and criticality, then it is ordered enough to maintain structure, but disordered enough so as to be adaptable to spatial and temporal changes. The mind exhibits these properties. Accordingly, the mind can be labeled under the SOC class of systems.

Unlike Gibsonian affordances, which are typically utilized in the study of organism-environment interactions, the theory of SOC can span investigations across varying scales of investigation. The broad applicability and mounting empirical evidence are why SOC, and not affordances, is RECN's guide to discovery. SOC provides a theoretical foundation from which to observe and explain phenomena that can be overlooked by other theoretical frameworks, including self-organization and behavior that is neither regular nor random. In the following section, I present a proof of concept to demonstrate the feasibility of applying DST as a unifying methodology and SOC as a productive guide to discovery across various scales of investigations between the mind sciences.

## Proof of concept

Many of the theories and methods discussed thus far have utilized DST. Although relatively new to the mind sciences, DST has an ever-growing track record of successful applications to the analysis of cognition and behavior. There remains debate as to the degree to which these methods can augment or replace more traditional methods in the cognitive, neural, and psychological sciences (e.g., Van Orden et al., 2003, 2005; Wagenmakers et al., 2005; Gray, 2012). This is not the debate I wish to enter here. I have discussed DST and its successes in order to support my claim that RECN can be a unifying framework across the mind sciences. What follows is an early attempt at a proof of concept for the application of DST and SOC across multiple scales in the mind sciences. I will attempt to start at the scale of single neurons and work my way up to interactions among body and world.

The basic treatment of individual neurons has been to conceive of them as on/off switches; they either fire or they don't. During the last decade or so, neurons and synapses have come to be understood as complex systems unto themselves (Izhikevich, 2007; Choquet and Triller, 2013; Wilhelm et al., 2014). The first part of my proof of concept is Izhikevich's (2010, p. 5067) model of individual synaptic activity:

$$I_{\text{synaptic}}(t)=\sum_{i}g_{i}(t)(E_{i}-v)$$

This equation is a model of synaptic activity, where *I* represents the sum of all input currents, including biological features such as time-varying conductance (*g_i_(t)*) and molecular chemical activity (*i* = NMDA, AMPA, GABA_A_, and GABA_B_). The above model is biologically realistic, meaning that it captures the activity of real synapses. Similar models of synaptic activity have explicitly demonstrated the success of utilizing SOC as a guide to discovery. Levina et al. (2007) demonstrated that by inputting biologically realistic parameters in another model of dynamical synapses in a spiking neural network, neuronal avalanches went from being occasionally observed to being typical behavior of the network indicative of self-organized critical behavior. In short, Levina and colleagues demonstrated that synaptic activity plays a crucial role in generating self-organized and critical behavior in biologically realistic neural networks.

The above model of synaptic activity can be nested within a model of single neurons (Izhikevich, 2010, p. 5063):

$$C\dot{v}=k\left(v-v_{\text{rest}}\right)\left(v-v_{\text{thresh}}\right)-u+I$$

and

$$\dot{u}=a[b\left(v-v_{\text{rest}}\right)-u]$$

This simple model of spiking neurons (*C*$\dot{v}$), along with a recovery variable ($\dot{u}$), captures such features as voltage resting (*v*_rest_) and threshold (*v*_thresh_) states. The previous set of equations can be simplified into a more general form as follows (Izhikevich, 2010, p. 5068):

$$\dot{v}=f(v,\;u)+g(t)\left[E(t)-v\right]+I$$

This model of neuron firing incorporates the synaptic term (*I*), such that all of the activity in the model of individual synaptic activity is nested within the model of single neuron activity. If, for example, all of the various molecular chemical activity involved in synaptic activity (e.g., NMDA, AMPA, GABA_A_, and GABA_B_) were not discovered yet, a biologically realistic model of single neuron activity could still be developed, with *I* still based on empirically obtained data, namely, recordings of synaptic activity. Now that the molecular chemical properties of synapses are known, the *I* term can be *zoomed in* on for more detailed information. Were the “details” of the *I* term not known, would it be said that the general model of single neuron firing was not a justified model of single neuron activations? Unless an investigator is a “smallest” and has a priori commitments to the notion that explanations are only had at a particular scale of investigation such as the molecular, then the general model of single neuron firing ought to be considered a justifiable explanation. The complexity of synapses (Wilhelm et al., 2014) can be captured by a single parameter in a model of a higher scale phenomenon such as single neurons. The same holds true as we scale up.

The next step of my proof of concept is to demonstrate that models of single neuron firing ($\dot{v}$) can be nested within models of networks of neurons. Izhikevich and Edelman (2008) simulated the behavior of total synaptic connections within the mammalian thalamocortical system. In the current model, each neuron is treated as a “compartment” that is connected to numerous other “compartments.” The model for total synaptic activity at each compartment is as follows (Izhikevich and Edelman, 2008, supporting information appendix, p. 11):

$$\begin{array}{l} I_{\text{syn}}=g_{\text{AMPA}}(v-0)+g_{\text{NMDA}}\frac{{\left[(v+80)/60\right]}^{2}}{1+{\left[(v+80)60\right]}^{2}}\left(v-0\right)\\ \;+g_{{\text{GABA}}_{\text{A}}}(v+70)+g_{{\text{GABA}}_{\text{B}}}(v+90)+I_{\text{gap}} \end{array}$$

The model above contains the *v* term from the previous equation. The model of compartment activity and their relationships, as captured by simulated fMRI/BOLD signals, can be captured by the single term ẏ (Izhikevich and Edelman, 2008, supporting information appendix, p. 12). Thus, the sum of all synaptic activity of all neurons within a voxel is modeled as:

$$\dot{y}=(I_{\text{syn}}(z))-y/500$$

This model is adapted from Izhikevich and Edelman (2008) and altered to explicitly include the *I*_syn_ term that itself incorporated the activity of the model of neuro firing captured by the $\dot{v}$ term in Izhikevich (2010). The *z* term refers to the number of compartments calculated. The remainder of the model remains unaltered.

Adapted from neuron models by Morrison and colleagues (Morrison et al., 2007), Rubinov et al. (2011) developed a model of network connections that exhibited SOC. Rubinov and colleagues found the presence of self-organized critical dynamics in their neurobiologically realistic hierarchically modular networks of integrate-and-fire neurons (such as those modeled by Izhikevich, 2010). Their network model is as follows (Rubinov et al., 2011, supplementary information, p. 3):

$$C\frac{dV}{dt}=C\frac{dy_{1}}{dt}=-gy_{1}+y_{2}-y_{3}$$

This model describes the integration of synaptic currents across all neural activity in a given area. In this model, *y*_1_ is equivalent to ẏ in the previous model of synaptic activity within a voxel. The other terms depict such features as membrane conductance (*g*), the timing of previous spikes (*t*), and experimentally obtained parameter values (*V*). For more information on the parameter values see Table 1 in Rubinov et al. (2011). This equation, along with the definitions of *y*_2_ and *y*_3_ (see Rubinov et al., 2011, supplemental text 1, p. 3), can be restated in matrix form:

$$\frac{d_{y}}{d_{t}}=A_{y}=\left[\begin{array}{ccc} -g/C & 1/C & -1/C\\ 0 & -1/t_{1} & 0\\ 0 & & -1/t_{2} \end{array}\right]\left[\begin{array}{c} y_{1}\\ y_{2}\\ y_{3} \end{array}\right]$$

Network activity can then be analyzed via connectivity matrices (Rubinov et al., 2011, pp. 2–6). Once network connections have reached this stage of modeling, it is then possible, in principle, to begin to couple groups of networks of neurons within a single brain, or neural activity between individuals. The following is an idealized model of two sets of networks, coupled in two dynamical equations that cannot be solved separately (inspired by Kelso and Tognoli, 2007, p. 52):

$$\frac{d_{y}}{d_{t}}=A_{y}=\left[\begin{array}{ccc} -\frac{g}{C} & \frac{1}{C} & -\frac{1}{C}\\ 0 & -\frac{1}{t_{1}} & 0\\ 0 & & -\frac{1}{t_{2}} \end{array}\right]\left[\begin{array}{c} y_{1}\\ y_{2}\\ y_{3} \end{array}\right](y-x)$$

and

$$\frac{d_{x}}{d_{t}}=A_{x}=\left[\begin{array}{ccc} -\frac{g}{C} & \frac{1}{C} & -\frac{1}{C}\\ 0 & -\frac{1}{t_{1}} & 0\\ 0 & & -\frac{1}{t_{2}} \end{array}\right]\left[\begin{array}{c} x_{1}\\ x_{2}\\ x_{3} \end{array}\right](x-y)$$

The reason these two equations cannot be solved separately is that they each contain parameters present within the other equation. Thus, any changes to *y* in the above equation will affect the below equation, and any changes to *x* in the below equation will affect the top equation. Epistemologically speaking, each equation can be treated as referring to entities independent of each other (e.g., two networks in one brain, two people having a conversation, etc.). However, from a systems perspective, because a change in one part of the system has causal and constitutive consequences for the other, the entities are ontologically a single system.

This sort of approach to explaining phenomena via sets of dynamical systems equations has also been used to understand animal-environment systems (see Beer, 1995; Chemero, 2009). Beer presented the following set of equations to model animal-environment systems (1995, p. 181):

$$\begin{array}{l} {\dot{X}}_{A}=A\left(X_{A};S\left(X_{E}\right)\right)\\ {\dot{X}}_{E}=E\left(X_{E};M\left(X_{A}\right)\right) \end{array}$$

As discussed above, such sets of dynamical systems equations allow us to model systems and capture the interrelated nature of the parameters. In Beer's example, a change in *S* (sensory input to the animal) will affect *M* (motor output), which will reciprocally affect *S*, and so on. Attempts are currently underway to model social interactions in a similar manner as the previous two sets of equations (e.g., Kelso et al., 2009; Dumas et al., 2010).

This section has been an attempt at proof of concept of RECN as an explanatory framework for the mind sciences. First, I have attempted to demonstrate the potential power of RECN to account for brain activity, cognition, and behavior by providing examples of research guided by the theory of SOC. Second, I have attempted to demonstrate that DST modeling can be used to capture SOC across multiple scales. A significant feature of DST modeling that I have drawn attention to is its nesting capacity. Synaptic activity can be “collapsed” into a single parameter, while maintaining biological realism, and embedding that parameter into a model of single neuron firing. Next, I demonstrated that neuron firing can itself be collapsed into a single parameter and embedded into models with varying purposes, such as capturing activity within a wide range of voxels and across networks. The examples to this point are empirically validated, with results explicitly guided by the theory of SOC (i.e., Levina et al., 2007; Rubinov et al., 2011).

Unlike the preceding steps, which have been validated by published experimental results, the next step of my proof of concept is speculative. Motivated by the ability to nest activities at one scale within parameters of models at another scale, and inspired by previous work on systems modeling (e.g., Beer, 1995; Kelso and Tognoli, 2007), I present an idealized model of coupled brain regions. It is in principle possible that the same sets of commitments to the theory of SOC and the methodology of DST can guide research at higher scales (e.g., macroscale brain networks, social interaction, etc.). Following this proof of concept, in the next section I explain how RECN can address the data deluge challenge facing the mind sciences.

## Addressing the grand challenges

If investigations of mind span brain, body, and environment, then the “grand challenges” (He et al., 2013) facing neuroscience are applicable to the other mind sciences as well. Since the goal of RECN is to connect investigations of mind at all scales, including the neural, then it must also meet these grand challenges. RECN currently has the methods and theory to address these challenges. The first grand challenge is to develop a common theoretical language for describing cognition and behavior across multiple scales of inquiry. The terminology and tools of DST can meet this challenge. A number of mind sciences have already been utilizing DST across various scales of inquiry: Cognitive science (e.g., Beer, 1995; Hausdorff, 2007; Chemero, 2009, 2013), neuroscience (e.g., Izhikevich, 2007; Loh et al., 2007; Balduzzi and Tononi, 2008), and psychology (e.g., Smith and Thelen, 2003; Van Orden et al., 2003, 2005; Ramos et al., 2011). DST's broad range of successful applications in the mind sciences motivates the claim that it is able to serve as a shared methodology. With a methodology in place, SOC can serve as a common theoretical perspective. As with DST, there is mounting evidence for the application of SOC as a theoretical perspective for the mind sciences across scales: Single neurons (e.g., Levina et al., 2007), meso- and macroscopic brain networks (e.g., Rubinov et al., 2011), and social behaviors (e.g., Ramos et al., 2011). Given the successful and wide-ranging application of DST and SOC in the mind sciences, it is possible that the first grand challenge is met. However, it can be argued that there is nothing special about DST or SOC, that is, there are other methodological and theoretical approaches that can do this work as well.

There are many thorny philosophical issues surrounding what the criteria are for the “best” scientific methods and theories. Those issues go far beyond the scope of the current work. It is true that other methods can be applied across the mind sciences. Pluralism is often a good thing (Chemero and Silberstein, 2008; Dale, 2008; Dale et al., 2009). In terms of addressing the two specific grand challenges facing the mind sciences, as long as the methods facilitate the application of a theory that gels with the empirical data, there is reason to be persuaded to apply RECN. RECN is a viable option for addressing the first grand challenge because the methods facilitate empirically justified applications of its theory. The more compelling reason, however, for utilizing RECN as a framework is its ability to address the second grand challenge.

The second grand challenge is to reduce big data to small data, or, to make the enormous amounts of data produced by the cognitive, neural, and psychological sciences comprehensible within and between fields. Another way of putting the challenge of reducing big data to small data is by asking the following question: How do we explain how enormous groups of interconnected neurons, brain networks, and social and environmental interactions produce wide repertoires of behaviors? Part of an answer lies in having a theory for interpreting and conceptualizing research data. SOC is theoretically and conceptually able to provide an explanation for how enormous groups of interconnected neurons, brain networks, and social and environment interactions produce wide repertoires of behaviors: Large numbers of highly, (often nonlinearly) connected parts can self-organize and give rise to behaviors interconnected with the local areas and global state of the system. These self-organized systems can be poised at critical states, thereby facilitating stability and instability, which are necessary for systems to be able to adapt to changes at the local and global levels of interaction. DST methods are able to capture these systems-based features.

As demonstrated above in the proof of concept, the ability to collapse large amounts of information into single parameters, while preserving biologically real facts, and embedding those parameters within models at various scales is part of what makes DST an especially powerful set of tools for the mind sciences. Although collapsing large amounts of information into single parameters may facilitate more readily understood explanations, a number of challenges arise with this method and systems-based approaches in general. By treating cognition and behavior as realized across multiple scales within a system, RECN is able to nest parameters and focus on one scale at a time. Scales are pragmatically delineated and examined based on particular research questions and from particular theoretical points of view (cf. Popper, 1963/2002). If no single scale occupies a privileged position, then researchers are faced with what Sporns calls the “parcellation problem” of identifying meaningful functional units (2012, p. 44). A tension arises between maintaining biological realism while facilitating explanations and understanding of complex systems phenomena (Favela, 2014).

Non-systems-based approaches to the mind sciences have been motivated by what Sporns refers to as the “Laplacian dream” (2012, p. 168). The Laplacian dream is the idea that explanations in the mind sciences are only had when activities can be fully predictable based on brute force, bottom-up strategies to capture the “positions and velocities” of every neuron and synapse. If the goal of the mind sciences is to understand the mind, then the “Laplacian approach” is the incorrect one to follow:

The point of building brain [mind, cognition, behavior, etc.] models… is to advance understanding of brain [mind, cognition, behavior, etc.] function, not creating *in silico* replicas that are as complex and incomprehensible as the real thing (Sporns, 2012, p. 168).

If understanding is the goal of the mind sciences, and if the mind is a multiscale systems phenomenon, then it is inevitable that models will be utilized to facilitate dimension reduction. With parameter reduction, “processes at smaller scales become part of compact descriptions of regularities at larger scales” (Sporns, 2012, p. 168). So, if the mind sciences are committed to the notions of no single privileged scale, and the goal is to understand the mind, then nested parameters in DST models are an excellent option.

Although a single neuron receives inputs via molecular exchanges at its synapses, a brain network receives input from countless neuronal connections, and a single organism receives innumerable environmental input, investigations at each scale need not produce explanations that include specific information from every other scale. DST provides accounts of phenomena in the form of models. These models have the power to maintain such explanatory virtues as control, prediction, and simplicity while providing accounts of systems with many degrees of freedom with simple models that eliminate the irrelevant degrees of freedom of that system in relation to the target of inquiry (cf. Batterman, 1998). Without dimension reduction, explanations of complex systems phenomena run the risk of considering too many factors relevant (cf. Lewis, 2000; Strevens, 2009) that can result in incomprehensible accounts.

RECN strives for models all the way down; as well as all the way up and side-to-side, for that matter. In other words, a model of a cognitive activity ought to, in principle, be able to allow a researcher to *zoom in* on each parameter. This is because neuron models are nested within brain network model parameters, which are in turn nested within models of the body, and within models of the organism-environment interactions. In this way, “Big Data” becomes “Small Data” in light of the capacity of models to be nested and to incorporate behavior relevant parameters.

## Conclusion

The fact that it is becoming more accepted that investigations of mind span the brain, body, and environment means that scientists must reckon with large amounts of data. RECN is a scientific framework for investigating the mind as a phenomenon that spans brain, body, and environment. RECN is inspired by RECS. Like RECS, RECN utilizes the methods of DST. Although not opposed to it, RECN is not committed to Gibsonian ecological psychology and the investigation of affordances as its theoretical basis. Instead, RECN is committed to the theory of SOC. The main reason for this difference is that RECN attempts to incorporate brain research whereas ecological psychologists do not. There is already evidence that the theory of SOC is applicable to neural, bodily, and social systems.

The methods of DST and the theory of SOC provide answers to the NSF's data deluge grand challenges. As a descriptive term for particular kinds of systems, SOC is broad enough (synapses, neurons, networks, etc.) but focused enough (systems that are self-organized, exhibit scale-free and self-similar properties, and exist in states that are neither regular nor random) to be useful. DST is scale-neutral and applicable to broad ranges of phenomena through modeling. These models have the capacity to facilitate dimension reduction. A consequence of this capacity is the ability to nest parameters within other models, depending on the target of investigation. For example, although a model of a neuron includes a number of parameters, when investigating brain networks, those parameters are causally unimportant to the network model, which treats the neuron as a single parameter itself. Details become nested within parameters without losing explanatory virtues such as control and predictability. In this way, the grand challenge of reducing big data to small data is met. Moreover, the grand challenges are met while preserving comprehensibility of complex systems phenomena without conceding biological realism.

### Conflict of interest statement

The author declares that the research was conducted in the absence of any commercial or financial relationships that could be construed as a potential conflict of interest.
